# Spermine Oxidase–Substrate Electrostatic Interactions: The Modulation of Enzyme Function by Neighboring Colloidal ɣ-Fe_2_O_3_

**DOI:** 10.3390/biom13121800

**Published:** 2023-12-15

**Authors:** Graziano Rilievo, Massimiliano Magro, Federica Tonolo, Alessandro Cecconello, Lavinia Rutigliano, Aura Cencini, Simone Molinari, Maria Luisa Di Paolo, Cristian Fiorucci, Marianna Nicoletta Rossi, Manuela Cervelli, Fabio Vianello

**Affiliations:** 1 Department of Comparative Biomedicine and Food Science, University of Padua, Viale dell’Università 16, 35020 Legnaro, Italy; graziano.rilievo@phd.unipd.it (G.R.); massimiliano.magro@unipd.it (M.M.); federica.tonolo@unipd.it (F.T.); alessandro.cecconello@unipd.it (A.C.); aura.cencini@studenti.unipd.it (A.C.); fabio.vianello@unipd.it (F.V.); 2 Department of Molecular Medicine, Laboratory Affiliated to Istituto Pasteur Italia, Fondazione Cenci Bolognetti, Sapienza University of Rome, Viale Regina Elena 291, 00161 Rome, Italy; lavinia.rutigliano@uniroma1.it; 3 Department of Geosciences, University of Padua, Via Gradenigo 6, 35131 Padova, Italy; simone.molinari@unipd.it; 4 Department of Molecular Medicine, University of Padua, Via G. Colombo 3, 35131 Padova, Italy; marialuisa.dipaolo@unipd.it; 5 Department of Sciences, University of Roma 3, Viale Guglielmo Marconi 446, 00146 Rome, Italy; cristian.fiorucci@uniroma3.it (C.F.); mariannanicoletta.rossi@uniroma3.it (M.N.R.); 6 International Polyamines Foundation ‘ETS-ONLUS’, Via del Forte Tiburtino 98, 00159 Rome, Italy

**Keywords:** nanoenzyme, spermine oxidase, enzyme activity, electrostatic interactions, ionic strength, enzyme nano-immobilization

## Abstract

Protein–nanoparticle hybridization can ideally lead to novel biological entities characterized by emerging properties that can sensibly differ from those of the parent components. Herein, the effect of ionic strength on the biological functions of recombinant His-tagged spermine oxidase (i.e., SMOX) was studied for the first time. Moreover, SMOX was integrated into colloidal surface active maghemite nanoparticles (SAMNs) via direct self-assembly, leading to a biologically active nano-enzyme (i.e., SAMN@SMOX). The hybrid was subjected to an in-depth chemical–physical characterization, highlighting the fact that the protein structure was perfectly preserved. The catalytic activity of the nanostructured hybrid (SAMN@SMOX) was assessed by extracting the kinetics parameters using spermine as a substrate and compared to the soluble enzyme as a function of ionic strength. The results revealed that the catalytic function was dominated by electrostatic interactions and that they were drastically modified upon hybridization with colloidal ɣ-Fe_2_O_3_. The fact that the affinity of SMOX toward spermine was significantly higher for the nanohybrid at low salinity is noteworthy. The present study supports the vision of using protein–nanoparticle conjugation as a means to modulate biological functions.

## 1. Introduction

Spermine oxidase (here abbreviated as SMOX; EC 1.5.3.16) is a dimeric FAD (flavin adenine dinucleotide)-containing enzyme involved in the polyamine catabolic pathway, oxidizing spermine into the reaction products of spermidine, 3-aminopropanaldehyde, and hydrogen peroxide in the presence of oxygen [1]. Besides its importance in regulating polyamine homeostasis in cells, it can represent an attractive option for enzyme therapy. As an example, the ability to generate toxic species [1] can be a potential key for circumventing the multidrug resistance (MDR) of tumor cells [2]. Indeed, SMOX belongs to a group of enzymes already tested for inducing cytotoxicity in human cancer cells, such as bovine serum amine oxidase (BSAO) [3]. Indeed, SMOX activity products, such as reactive oxygen species, H_2_O_2_, and 3-aminopropanal aldehyde, are able to evoke cellular damage, leading to several pathologies [4].

Unfortunately, the applicability of enzymes as drugs in real-world scenarios is hampered by limitations, such as very low membrane permeability and intrinsic instability [5]. Nanomaterials are currently widely studied as an innovative delivery strategy for biomolecules, drugs, and enzymes into cells, and novel smart nanovehicles have been proposed for targeting diseased tissues [6,7].

In the last decade, the hybridization of nanoparticles and enzymes relied on a plethora of core materials [8] and binding strategies [9]. Although the influence of enzyme immobilization on structure and activity is hardly predictable and can lead, at worst, to protein denaturation and loss of biological function [10], the enhancement of enzyme activity is realistic as well and seems to depend on the proper protein–nanoparticle combination [11,12]. In this view, a number of examples were proposed for the immobilization of enzymes, leading to increased stability [13], enhanced activity, specificity, and selectivity compared to soluble enzymes [14].

Overall, protein–nanomaterial interactions are extremely complex and far from being fully comprehended, requiring suitable nanomaterial surfaces to harbor the enzyme, as well as delicate binding methods to avoid the well-known immobilization-related risk of protein denaturation. In the limitless arena of nanomaterials, among the choice of available iron oxide nanomaterials, peculiar superparamagnetic nanoparticles constituted of stoichiometric maghemite (ɣ-Fe_2_O_3_) have emerged as versatile platforms for producing self-assembled and functional nano-bio-conjugates. These nanoparticles, called surface active maghemite nanoparticles (SAMNs), are characterized by high colloidal stability in the absence of any superficial modification or coating derivatization and a unique surface chemistry [15]. This endows SAMNs with the ability to bind proteins in a highly selective way, and, most importantly, macromolecules with affinity for SAMNs can readily interact with the nanoparticle surface without dramatic structural alterations [15]. On the other hand, even minimal structural rearrangements occurring upon protein docking on SAMNs can result in a relevant change in immobilized enzyme catalytic activity [16].

In the present work, by coupling His-tagged SMOX and pristine nanoparticles SAMNs, a catalytically active enzyme–nanoparticle hybrid (SAMN@SMOX) was fabricated and characterized.

Herein, along with the intrinsic features of the nanomaterial core, including superparamagnetism and fluorescence [17], the enzymatic cargo (SMOX) displayed new biological features as a consequence of direct immobilization. In particular, the SAMN@SMOX hybrid displayed a considerably higher affinity toward its substrate. These differences were attributed to conformational alterations of the enzyme as evidenced through the use of circular dichroism spectroscopy and FTIR and by the zeta potential of the final nanohybrid.

## 2. Materials and Methods

### 2.1. Reagents

All reagents were purchased at the highest commercially available purity and were used without further purification. His-tagged (HT) SMOX (mouse spermine oxidase) expressed in *Escherichia coli* was purified according to [18]. The enzyme (Mr = 68 kDa per monomer, 136 kDa the holoenzyme) was obtained at a concentration of 1.42 µg/µL in 10 mM HEPPS buffer (N-[2-hydroxyethyl]piperazine-N’-[3-propanesulfonic acid]) at pH 8.0, and stored at −20 °C. Surface Active Maghemite Nanoparticles (SAMNs) were produced in-house following a protocol proposed by Magro et al. (2012) [19]. HEPPS buffer, sodium chloride (NaCl), di-thiothreitol (DTT), N,N-dimethyl-aniline (DMA), 4-amino-antipyrine (AMP), horseradish peroxidase type II (HRP, 179 units/mg solid) and spermine (Spm) were purchased from Sigma-Aldrich at high-grade purity. A series of Nd-Fe-B magnets (N35, 263–287 kJ/m^3^ BH, 1170–1210 mT flux density by Power magnet—Germany) was used to magnetically recover the nanoparticles.

### 2.2. Instrumental Analysis

Protein fluorescence was assessed by using a Varian Cary Eclipse Fluorescence Spectrometer (Agilent, CA, USA). The instrument settings were as follows: λ_ex_ 280 nm, λ_em_ 300–500 nm, slit 10 nm/20 nm, medium scan rate acquisition (600 nm/min). The volume of the samples was 400 µL in a quartz cuvette. For protein quantification by fluorescence, a calibration curve was built with concentrations ranging from 0 to 200 mg/L in 10 mM HEPPS buffer at pH 8.0 (Figure S1 in Supplementary Material). The hydrodynamic radii and zeta potential values of bare SAMNs and of the nanohybrid were measured via dynamic light scattering (DLS) using a Zetasizer Nanoparticle analyzer ZEN3600 (Malvern Instrument, Malvern, UK). Both measurements were carried out with naked SAMNs and SAMN@SMOX at 50 mg/L concentration in 1 mM HEPPS pH 8.0 at room temperature. The enzyme activity was assessed following the kinetic assay described by Stevanato et al. [20]. Briefly, SMOX was incubated in the presence of 3 mM N,-N dimethyl-aniline (DMA), 4 mM 4-amineanitpyrine (AMP), 5 U/mL horseradish peroxidase (HRP), and Spm as substrate in a 20 mM HEPPS buffer at pH 8.0, at 28 °C. The hydrogen peroxide produced by the two-step reaction was continuously monitored by the change of absorbance at 544 nm, using a molar extinction coefficient (ε) of 1.25 × 10^4^ M^−1^cm^−1^. Kinetic assays were performed with increasing concentrations of the substrate (from 0.01 to 1.00 mM spermine), using 5 mg/L of soluble enzyme and 0.25 g/L SAMN@SMOX. The kinetic parameters were determined according to the Michaelis–Menten model. The enzyme kinetic characterization was carried out with a VICTOR X4 2030 Multilabel Reader (Perkin Elmer, Waltham, MA, USA) with a 96-well Iwaki microplate (Asahi Techno Glass, Tokyo, Japan). The production of the colored dye was monitored for 1 h, and the initial velocity (v_0_) was extrapolated in the linearity range comprised between 10 min and 25 min and plotted according to the Michaelis–Menten model. As controls, measurements in the absence of substrate were considered. Fourier transform infrared (FTIR) analysis of native enzyme, bare SAMNs, and SAMN@SMOX was performed using an IR Affinity-1S spectrometer (Shimadzu Corp., Kyoto, Japan) equipped with a diamond ATR analyzer and LabSolutions IR software (Shimadzu Corp., Kyoto, Japan, version 2.21, accessed on 25 April 2018). The scanning range was between 500 and 4000 cm^−1^ with a resolution of 4 cm^−1^ and 300 accumulated scans. Quantitative analysis of the native enzyme secondary structures and SAMN@SMOX hybrid was based on a curve fitting of the amide I band, according to Hebia and co-authors [21]. The structure content was quantified via band deconvolution using a Gaussian model considering the following secondary structure motifs: β-sheet (1637–1610 cm^−1^), random coil (1648–1638 cm^−1^), α-helix (1660–1650 cm^−1^), β-turn (1680–1660 cm^−1^) and β-antiparallel (1692–1680 cm^−1^). Circular dichroism spectra were acquired by using a Jasco J-800 instrument (Jasco Int. Co., Tokyo, Japan) in 10 mM HEPPS, pH 8.0 in a quartz cuvette (p.l. 0.2 cm). The analysis of the CD spectra was carried out using BeStSel (Beta Structure Selection, version 3.0., accessed on 18 July 2023), which is a free online software tool found at https://bestsel.elte.hu/index.php. Transmission electron microscopy (TEM) micrographs were acquired by using a Jeol JEM-2010 microscope (Jeol Ltd., Tokyo, Japan) operating at 200 kV with a point-to-point resolution of 1.9 Å. Before measurements, the samples were dispersed in ethanol and the suspension was treated using ultrasound for 10 min. A drop of dilute suspension was placed on a carbon-coated copper grid and allowed to dry via evaporation at room temperature.

The amino acidic sequence of SMOX was retrieved from the RCSB Protein Data Bank. Since the PDB code of mouse SMOX is not available, the crystal structure of the human SMOX was selected (PDB code: 7OXL). This was carried out knowing that the sequences of the two structures are equal at a 94.23% level (a comparison was made with the SWISS-MODEL Repository [22]). The selected PDB code was then used for the image processing with PyMOL (The PyMOL Molecular Graphics System, Version 2.0 Schrödinger, LLC, New York, NY, USA).

The dependence on ionic strength (I) of kinetic parameters (k_cat_, K_M_ and k_cat_/K_M_) of soluble and SAMN immobilized SMOX was studied in 10 mM HEPPS at pH 8.0 by adding 5–25 mM NaCl. The kinetic data were analyzed according to the Debye–Hückel equation [23]:

(1) $$logk=logk_{0}+2CZ_{a}Z_{b}{\left(I\right)}^{\frac{1}{2}}$$

where k is the kinetic parameter (k_cat_, K_M_ and k_cat_/K_M_), Z_a_ and Z_b_ are the charges of the interacting species, k_0_ is the value of the kinetic parameter at I = 0, and constant C is assumed to be 0.5 M^−1/2^ at 22 °C, in water [24]. A least-squares analysis was performed with commercial graphic software (SigmaPlot 10.0 program, Jandel, Scientific, Valencia, Spain). The values of the best-fit parameters and the standard error of the mean value (SEM) are reported. All determinations were performed at least in triplicate.

## 3. Results

### 3.1. Chemical–Physical Characterization of the SAMN@SMOX Hybrid

**Figure 1:**
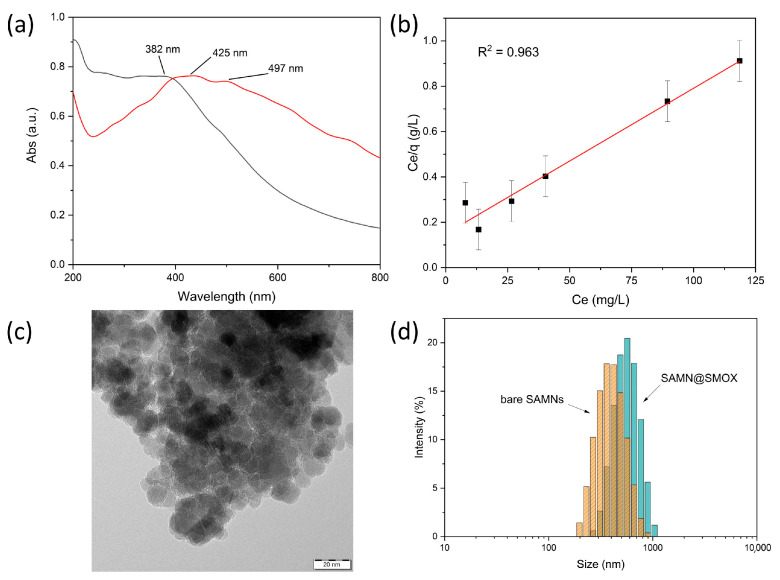
Chemical–physical and morphological characterization of SAMN@SMOX. Comparison of the UV-Vis spectra of naked SAMNs (black line) and SAMN@SMOX: (**a**) linearized Langmuir isotherm of the SMOX binding onto SAMNs; (**b**) linear Langmuir model; (**c**) TEM analyses of the SAMN@SMOX; (**d**) DLS measurements with the statistical fitting according to the LogNorm function, orange bars for bare SAMNs and blue bars for SAMN@SMOX.

Aiming at the development of a novel biologically active nano-hybrid, a simple self-assembly approach was used for the direct interaction of SMOX with naked SAMNs. The protein-strong chelating moieties, i.e., the His-tags present in the recombinant enzyme, were used to anchor SMOX to the SAMN surface according to the following rationale. At the physical boundary of maghemite nanoparticles, the crystal is interrupted, and, as a consequence, the surface exposes a distribution of iron (III) sites to the milieu, which are not entirely coordinated. Therefore, ligand binding is thermodynamically favored as it induces the restoration of the aforementioned dangling bonds [15]. This phenomenon is known as surface reconstruction, and generally, it is accompanied by a red shift of the nanoparticle absorption spectrum [25]. Optical transitions are the consequence of charge transfer between the donating organic modifier (SMOX in the present case) and the conduction band of metal oxides (SAMNs). The fact that surface reconstruction is a characteristic of metal oxide systems displaying high crystallinity, dimensions below 20 nm and, actual colloidal stability is noteworthy. In this view, SAMNs represent an elective paradigm, and the aforementioned red shift emerged as a common trait in our previous studies, including nanoparticle hybridization with proteins [19]. In Figure 1a, the integration of SMOX with SAMNs induced a red shift of the absorption maximum of about 40 nm and the appearance of a shoulder at around 500 nm, confirming the expected coordinative nature of the SAMNs–SMOX interaction. Furthermore, the binding of SMOX onto the SAMN surface was studied through the use of adsorption isotherm models, according to the work of Giles [26] and Langmuir [27]. The binding reaction was performed in 10 mM HEPPS buffer at pH 8.0 at a constant SAMN concentration (500 mg L^−1^) and SMOX concentrations ranging from 5 to 200 mg L^−1^ under gentle agitation for 2 h at 4 °C. In order to release loosely bound SMOX, the hybrids were magnetically separated and washed several times with incubation buffer. In order to estimate the concentration of bound enzyme, the SMOX concentration in the supernatants of the hybridization and washing steps was compared to the initial enzyme concentration. Protein quantification was carried out via spectrofluorometric measurements, as described in the materials and methods section.

The Giles model [26] is a useful preliminary approach that considers the trend of the curve of the bound ligand (Q) against the free ligand in solution at the equilibrium (C_e_). In Figure S2, the SAMN–SMOX system displayed saturation behavior, indicating the successful integration of the biological macromolecule to the magnetic core and prompting that once the first shell is completed, no further protein adsorption to SAMNs can occur. On these bases, the Langmuir isotherm model represents a suitable model for a more in-depth study of the development of a monomolecular core–shell system. Actually, one fundamental assumption of the Langmuir model is the formation of a single adsorbate monolayer [27]. The following linearized form of the Langmuir isotherm was adopted:

(2) $$\frac{C_{e}}{Q}=\frac{1}{Q_{max}K_{L}}+\frac{1}{Q_{max}C_{e}}$$

where Q is the loading capacity (mg g^−1^, namely mg protein on g nanoparticles) at a specific protein equilibrium concentration (C_e_ is expressed in mg L^−1^), Q_max_ is the maximum loading capacity (expressed as mg g^−1^), and K_L_ is the Langmuir stability constant (expressed in mL mg^−1^). Q_max_ and K_L_ were calculated from the slope and the intercept of the linear C_e_/Q vs. C_e_ plot.

The fact that the Langmuir isotherm properly fitted SMOX binding is noteworthy (R^2^ = 0.963, Figure 1b), confirming the formation of a mono-molecular shell on the SAMN surface. The theoretical maximum loading capacity, Q_max_, resulted in 155.8 ± 13.6 mg SMOX per g of SAMNs, which is fully in harmony with previously reported single-layer core–shell systems obtained via the direct hybridization of SAMNs with large polypeptidic molecules [16,19]. Furthermore, the calculated Langmuir constant, K_L_, resulted in 43.1 ± 10.4 mL/mg, which is again in very good agreement with stable Langmurian nano-bio-conjugates [19].

Taking into consideration the loading capacity (*Q_max_*), the theoretical number of SMOX molecules per single SAMN was calculated using the following equation:

(3) $$\frac{number\;of\;SMOX}{SAMN}=\frac{Q_{max}NA\;V\;d_{\gamma -Fe2O3}}{M}$$

where NA is the Avogadro number, M is the molar mass of the SMOX dimer (136 kDa, vide supra), V is the volume of a single nanoparticle, calculated by using a simple approximation of a SAMN to a sphere with an average diameter of 11 nm, and d_ɣ-Fe2O3_ is the density of maghemite (4.8 g cm^3^). The product of the last two terms is the mass of a single SAMN. The ratio resulted in 2.4; hence, it can be concluded that a monolayer could likely comprise from 2 to 3 enzyme molecules per nanoparticle.

The morphological and hydrodynamic features of SAMN@SMOX were examined using transmission electron microscopy (TEM) and dynamic light scattering (see Section 2). TEM micrographs of SAMN@SMOX (Figure 1c) witnessed the formation of core–shell hybrids constituted of a single, well-preserved magnetic core embedded in a less electron-dense organic envelope. However, the relatively contained thickness of the carbonaceous phase, measuring around 2 nm, can be ascribed to TEM sample preparation. Furthermore, the zeta potential (ζ) measurements were carried out and under the current conditions (see Section 2), the ζ value of the bare nanoparticles resulted in +6.7 ± 1.6 mV (conductivity = 0.072 mS/cm at 25 °C). The remarkable colloidal stability of water suspensions of SAMNs has been extensively commented on in several previous publications, and it is mirrored by an extremely high ζ for naked iron oxide nanoparticles, standing well above +30.0 mV. Here, the low zeta potential value registered can be likely attributed to the pH of the medium used for the analysis (pH = 8.0). The ζ value of SAMN@SMOX was −19.7 ± 0.5 mV (conductivity = 0.057 mS/cm at 25 °C), which is noteworthy. It should be considered that an aqueous suspension of a nanomaterial possessing a ζ within the 20–30 mV range can be classified as stable for either positive or negative values. The latter is a suitable characteristic in terms of future in vitro and in vivo investigations. The analysis of the hydrodynamic radii is reported in Figure 1d. For unmodified SAMNs, the hydrodynamic size resulted in 432.6 ± 56.9 nm, which is exceptionally large in comparison to that measured in water suspension (Figure 1d, orange bars). Again, this can be ascribed to the aggregation processes at the pH of the milieu employed in the self-assembly reaction and used in the DLS analysis. Although the apparent discrepancy between the TEM and DLS measured sizes could, in principle, point to the partially aggregated state of the nano-hybrids [28], it is more likely that the size overestimation when using DLS is due to the hydration shell and counter-ion clouds around the nanohybrids, which is in line with similar reports [29,30]. Indeed, DLS actually determines the hydrodynamic size of nanoparticles, while it is important to consider that a hydration shell cannot be observed under the vacuum conditions of TEM. SAMN@SMOX showed a hydrodynamic diameter of 787.7 ± 48.3 nm (Figure 1d, blue bars). The magnitude of the measured hydrodynamic radius is comparable with previously reported core–shell nanostructures constituted by a single SAMN core and a protein mono-molecular layer [16].

**Figure 2:**
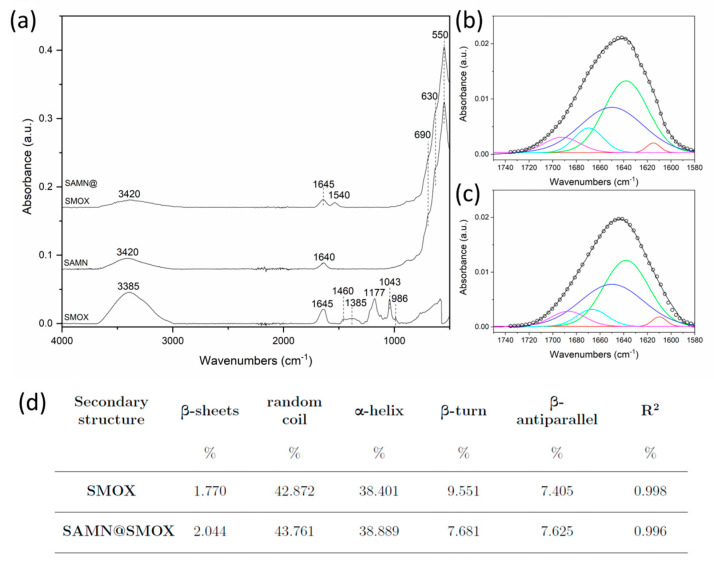
(**a**) FT-IR spectra of SMOX, naked SAMNs and SAMN@SMOX complex. (**b**,**c**) Deconvolution of amide-I band of SMOX and SAMN@SMOX complex, respectively. Experimental amide-I band (black line), Gaussian fitting curve (black dots), β-sheet (red line), random coil (green line), α-helix (blue line), β-turn (light blue line) and β-antiparallel (purple line). (**d**) Secondary structure contents of native SMOX and of the SAMN@SMOX hybrid according to the deconvolution of the FTIR amide-I band.

Fourier transform infrared spectroscopy (FTIR) was used to investigate the occurrence of possible structural alterations to the enzyme upon direct immobilization on the SAMN surface. As visible in Figure 2a, the SAMN@SMOX complex evidence two main bands at 1645 and 1540 cm^−1^ corresponding to SMOX amide-I and amide-II bands, thus confirming the successful immobilization of the enzyme. All the other observable bands in the FTIR profile of the SAMN@SMOX complex can be ascribed to the nanoparticle core. In particular, the peaks at 550, 630 and 690 cm^−1^ are the Fe-O stretching vibrations, while that at 3420 cm^−1^ refers to OH stretching.

Interestingly, the amide-I band of SMOX did not experience a shift in position nor a visible change in the shape upon binding, thus suggesting the preservation of the structure of the native enzyme. In order to investigate the secondary structure conformation of SMOX in depth and quantify even negligible structural changes upon binding, the amide-I band was subjected to deconvolution (Figure 2b,c). The contributions of all the structural components obtained via the analysis are reported in Figure 2d. The deconvolution clearly shows that the interaction between SMOX and SAMNs slightly affected the enzyme structure. The whole structural components highlight changes in the range of 0.2–2%, thus suggesting that the enzyme was unaffected upon complexation.

**Figure 3:**
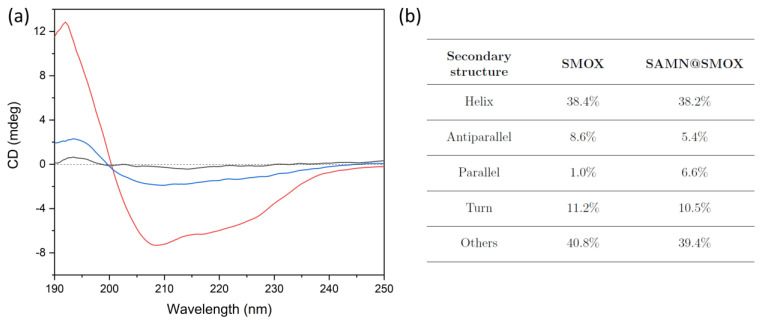
(**a**) Circular dichroism spectra of SMOX (red, 0.075 g L^−1^), naked SAMNs (black, 0.5 g L^−1^) and SAMN@SMOX (blue, 0.5 g L^−1^) in 10 mM HEPPS at pH 8.0. (**b**) Secondary structure contents of native SMOX and of the SAMN@SMOX hybrid according to circular dichroism.

In order to shed more light on the possible structural modification of SMOX upon immobilization on SAMNs, both enzyme forms were characterized via circular dichroism (CD). The CD spectrum of parent SMOX showed a positive peak at 195 nm and a negative broad band, approximately centered at 220 nm, which is common in proteins (Figure 3, red line) [31]. The same features were observed in the CD spectrum of the SAMN@SMOX hybrid (Figure 3, blue line), providing additional evidence of the self-assembly of the SAMN@SMOX nano-bio-conjugate, as well as of the preservation of the overall structure of the native enzyme. It is worth mentioning that based on the author’s knowledge, even minor conformational changes can result in drastic modifications in terms of catalytic behavior [16].

### 3.2. Comparison of the Activity of Native SMOX and of SAMN@SMOX Hybrid

**Figure 4:**
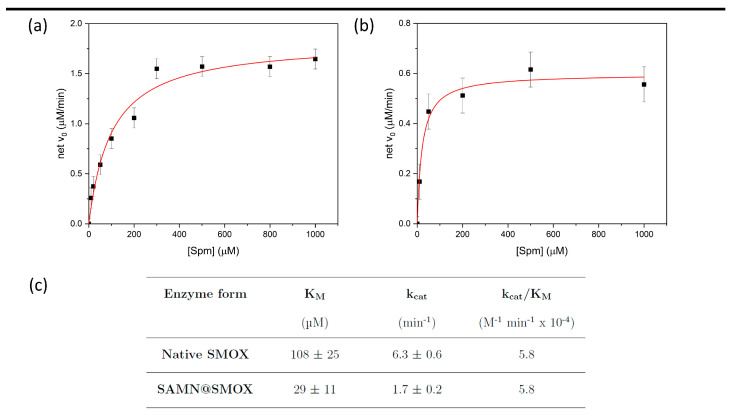
Kinetic study of the native SMOX and of SAMN@SMOX. Michaelis–Menten curves of native SMOX (**a**) and of SAMN@SMOX (**b**); (**c**) kinetic parameters of SMOX and of SAMN@SMOX.

The kinetic parameters (i.e., K_M_, k_cat_ and k_cat_/K_M_) of native and nano-immobilized SMOX were determined through the use of the spectrophotometric assay described by Stevanato et al. [20] and compared according to the Michaelis–Menten model, as shown in Figure 4a,b. The kinetic parameters obtained are reported in Figure 4c.

The fact that the Michaelis–Menten constant showed a significant decrease upon SMOX nano-immobilization is worthy of note. This is not a trivial outcome, revealing the enhanced affinity of the immobilized SMOX for spermine. In contrast, the k_cat_ value exhibited by the SAMN@SMOX hybrid, even if it was lower than that of the native enzyme, indicates that naked SAMNs disclose a favorable local environment for enzyme harboring. Interestingly, the catalytic efficiency (k_cat_/K_M_) did not change upon immobilization, indicating that the activity of SMOX at low substrate concentration was not affected by SAMNs. This result suggests the feasibility of the application of the SAMN@SMOX hybrid in terms of the preservation of enzyme activity under physiologic conditions.

In this view, the hybrid was re-used at least three times and its catalytic activity was preserved (100%) after 3 months of storage at 4 °C, highlighting the robustness of nano-immobilized SMOX.

**Figure 5:**
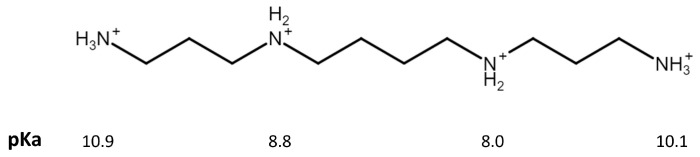
Spermine chemical structure. Evidencing the four amino groups and the corresponding pKa values used for calculating the electrical charge of the polyamine [36].

Previous studies [32,33] have shown that the SMOX active site contains polar residues (Ser527, Tyr482, Gln200, His82 and Glu224), which play a key role in the SPM–SMOX interaction. In particular, these residues are involved in the positioning of the substrate into the active site by electrostatic/polar interaction (such as between Glu224 and the positively charged N14 of SPM), consequently affecting the rate of the chemical step (represented by the catalytic constant). Thus, to obtain information on the electrostatic interactions involved in the activity of soluble and SAMN immobilized SMOX, and, most importantly, on the effect of the iron oxide nanoparticle on substrate recognition and oxidation by the immobilized enzyme, the dependence of the kinetic parameters k_cat_/K_M_, k_cat_ and 1/K_M_ on ionic strength (I) was studied using spermine as a substrate. The K_M_ value, according to the Michaelis–Menten model, is defined by the contribution of different kinetic constants, including k_cat_ [34], and, under particular conditions, it represents the dissociation constant of the substrate–enzyme complex. Consequently, to evaluate the effect on the association constant of the SMOX–SPM complex, 1/K_M_ values should be considered. Measurements were carried out at pH 8.0, and at this pH value, the calculated electrical charge of spermine is +3.34 [35], see Figure 5.

Moreover, different from previous kinetic characterizations of SMOX [33], the measurements were carried out in 10 mM HEPPS at pH 8.0 (I = 5 × 10^−3^ M) [37], and ionic strength was varied via the addition of NaCl (5–25 mM). The results were analyzed according to the Debye–Hückel equation [23], as described in the Methods section. The plots of log(k_cat_) vs. I^1/2^, log(1/K_M_) vs. I^1/2^ and log(k_cat_/K_M_) vs. I^1/2^ of SMOX and SAMN@SMOX showed roughly linear dependences, indicating the important role of electrostatic interactions in recognition and in the catalytic steps of both enzyme forms (Figure S3). Indeed, from the slopes of the above-mentioned plots (2C·Z_enz_·Z_sub_) reported in Table 1, it is possible to estimate the product of interacting charges (Z_enz_·Z_sub_) during enzyme activity on spermine, being the 2C factor of Equation (1) approximately equal to 1.

As regards soluble SMOX, a strongly negative value of the slope of the log(k_cat_) vs. the square root of ionic strength plot was found (2C·Z_enz_·Z_sub_ ≈ −5.6), indicating that the rate of the catalytic steps depends on the interaction of opposite charges, and, considering the charge of spermine (Z_sub_ = +3.34), the enzyme should contribute about two negatively charge residues, which is in agreement with previous studies [33]. In contrast, in the case of SAMN@SMOX on spermine, the dependence of the catalytic constant on I^1/2^ showed a lower value in terms of interacting charge products (2C·Z_enz_·Z_sub_ ≈ −1.6). Possibly, the slight modification of enzyme structure upon immobilization on SAMNs observed by using circular dichroism affected the catalytic steps (k_cat_ of the soluble enzyme is higher than that of immobilized SMOX), changing the role played by the electrostatic interactions.

As regards the slope of the log(1/K_M_) vs. the I^1/2^ plot of soluble SMOX, its positive values (2C·Z_enz_·Z_sub_ ≈ +7.7) suggest the involvement of about two positive charges in the enzyme active site involved in the control of the substrate-active site recognition process. In contrast, in the case of the SAMN@SMOX hybrid, the 2C·Z_enz_·Z_sub_ product was negative (2C·Z_enz_·Z_sub_ ≈ −3.9), indicating an important reduction in enzyme affinity for spermine with increasing ionic strength. This effect can be attributed to reduced electrostatic attraction between the positively charged substrate and the nanoparticle-immobilized SMOX (zeta potential, ζ = −19.7 ± 0.5 mV) produced by the increasing electrolyte concentration. Finally, the effect of ionic strength on the catalytic efficiency (k_cat_/K_M_) of SMOX and SAMN@SMOX was considered. The k_cat_/K_M_ parameter represents the apparent second-order kinetic constant of the enzyme–substrate reaction, namely the kinetic constant defining the enzyme activity at low substrate concentrations ([S] << K_M_). The calculated 2C·Z_enz_·Z_sub_ product corresponding to the slope of the log (k_cat_/K_M_) vs. I^1/2^ was ≈+1.9 in the case of soluble SMOX and ≈−5.9 for the SAMN@SMOX hybrid. Considering the fact that at low ionic strength (I = 5 × 10^−3^ M in 10 mM HEPPS at pH 8.0), the catalytic efficiency (k_cat_/K_M_) of SMOX and SAMN@SMOX assumed identical values (see Figure 4c), enzyme binding to nanoparticles drastically modified the electrostatic interactions between SMOX and its substrate. The reduction in the k_cat_/K_M_ kinetic constant with ionic strength can be interpreted as the shielding of electrostatic attraction between SAMN@SMOX (zeta potential, ζ value of SAMN@SMOX = −19.7 ± 0.5 mV) and spermine as a substrate (Z_sub_ = +3.34).

**Figure 6:**
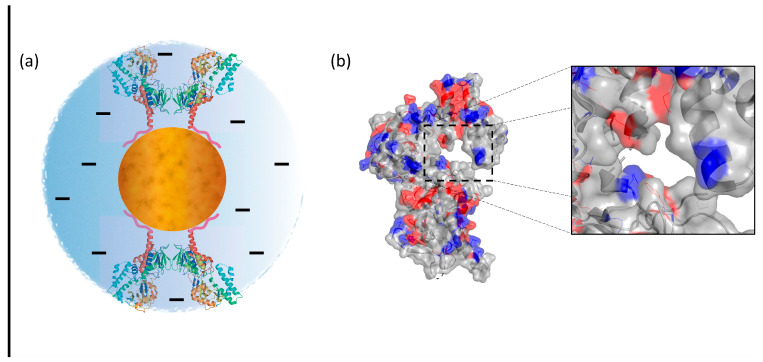
(**a**) Pictorial representation of SMOX–SAMN interaction with the computation model of the protein (represented as alfa, beta and random-coil structures) bridged to the nanoparticle (orange sphere) surface by exemplified His-tag moieties (pink-colored tails). (**b**) The N-terminus side pointing the solvent. Inset: the exposed catalytic cleft. The surface color scale was obtained using PyMOL, selecting the negative, positive and uncharged amino acids. Blue: ncharged amino acids (Aspartic acid and Glutamic acid), Red: positively charged amino acids (Lysine, Histidine and Arginine) Gray: uncharged amino acids.

Computational simulations of interfaces are widely considered reliable methods to understand nanomaterial–biomolecule interactions [38]. Herein, in order to identify the macromolecule region used by SMOX to spontaneously anchor onto the SAMN surface and its spatial positioning in the SAMN@SMOX hybrid, molecular simulations using a protein representation software were performed. Actually, the steric orientation is of fundamental importance for the availability of the enzyme active site. The crystal structure of SMOX (PDB code: 7OXL) was obtained from the Protein Data Bank and processed using PyMOL (The PyMOL Molecular Graphics System, Version 2.0, see Section 2). The recombinant protein exposes the His-tag moieties on the opposite side with respect to the catalytic site, namely at the C-terminus [38]. In this view, it is important to recall the strength of the His-tag groups as Fe^3+^ chelators, making the C-terminus an elective side for the docking of SMOX onto the nanoparticle surface. As reported elsewhere, proteins readily displaying interacting regions do not need to adapt their structure to maximize contact with the nanoparticles [15]. This fact plausibly explains the preservation of SMOX’s three-dimensional structure, which is necessary but not sufficient requisite for the enzyme to exert its biological activity. In Figure 6a, the 3D conformation of SMOX can be observed as well as a pictorial representation of its interaction with SAMNs. The fact that the latter would force the protein to expose the catalytic site to the milieu is noteworthy, representing a mandatory condition for substrate recognition. Finally, the negative nano-environment generated by the hybrid likely influences the electrostatic interactions between the charged amino-acid in the catalytic pocket and the substrate (Figure 6b).

## 4. Discussion

The use of enzymes as drugs is hampered by several factors, including the possible loss of catalytic activity and low bioavailability. Despite the well-known risk related to enzyme binding to solid surfaces, enzyme–nanomaterial hybridization is believed to provide a real chance of overcoming these limitations. Most importantly, there is an increasing consciousness that the proper enzyme-nanoparticle combination can also lead to unpredictable novel biological features that can be strategically employed in real-world scenarios. In the present work, SMOX was hybridized with peculiar iron oxide nanoparticles, merging supermagnetism and intrinsic fluorescence with unique colloidal stability. Indeed, the as-obtained SAMN@SMOX nanohybrid represents an interesting example of the possible modulation of the functions of an enzyme due to protein–nanoparticle coupling, ideally leading to a pseudo-novel biological entity., Besides showing a slightly reduced catalytic activity in comparison to the native enzyme, it is worth noting that the bioactive cargo revealed its own distinctive behavior related to its response to ionic strength. In this view, soluble SMOX was subjected for the first time to an extensive kinetic characterization in an interval of medium salinity ranging from 5 to 25 mM NaCl, illuminating the fact that SMOX–spermine interplay is ruled by electrostatic interactions. These interactions, when the enzyme is in its nano-immobilized form, are influenced by ionic strength in a completely different manner. To summarize, the main results on the effect of ionic strength evidenced that the physical interactions in the SMOX active site are affected by ionic strength involving positive charges of SPM and soluble SMOX. On the other hand, when the enzyme is immobilized on nanoparticles, the presence of the SAMN surface and slight SMOX structural modifications determine the modification of the effect of the electrostatic interactions between the enzyme and its substrate. In this case, the interactions involve positive charges of SPM and negatively charged nano-environment generated by SAMN@SMOX (vide supra), significantly improving the substrate–enzyme recognition steps.

## 5. Conclusions

SMOX has great importance due to its involvement in the polyamine catabolic pathway and, due to its biological function, it can be strategically employed in enzyme therapy. The present work, besides suggesting the feasibility of the application of the SAMN@SMOX hybrid in terms of preserving enzyme activity under physiologic conditions, encourages nascent awareness of the often-unpredictable benefits derived from enzyme–nanoparticle hybridization. In particular, minor structural modifications of the enzymatic cargo and of the nano-environment that SAMN@SMOX hybrid exposes to the solvent emerged as potential key factors concerning the modulation of SMOX function.

## Figures and Tables

**Table 1:** Slopes of the log(k_cat_) vs. I^1/2^, log(K_M_) vs. I^1/2^ and log(k_cat_/K_M_) vs. I^1/2^ (that is 2C·Z_enz_·Z_sub_ products) of native SMOX and SAMN@SMOX, where k_cat_ is the catalytic constant, K_M_ is the Michaelis–Menten constant and the k_cat_/K_M_ ratio is the catalytic efficiency.

	2C·Z_enz_·Z_sub_		
	k_cat_	1/K_M_	k_cat_/K_M_
Native SMOX	−5.6	+7.7	+1.9
SAMN@SMOX	−1.6	−3.9	−5.9

## Data Availability

Data is contained within the article or Supplementary Materials.

## Supplementary Materials

The following supporting information can be downloaded at: https://www.mdpi.com/article/10.3390/biom13121800/s1; Figure S1: fluorescence calibration curve of the enzyme spermine oxidase in 10 mM HEPPS pH 8; Figure S2: Giles isotherm of the SMOX binding onto SAMNs; Figure S3: dependence of kinetic parameters of soluble SMOX and SAMN@SMOX on ionic strength.
