# Supplementary material for: Spermine Oxidase–Substrate Electrostatic Interactions: The Modulation of Enzyme Function by Neighboring Colloidal ɣ-Fe2O3
Source: Biomolecules. 2023 Dec 15;13(12):1800. doi: 10.3390/biom13121800 (PMC10742170; doi:10.3390/biom13121800)
Supplement: Supplementary file 1 [file biomolecules-13-01800-s001.zip › biomolecules-2731068-supplementary.pdf]

Spermine oxidase-substrate electrostatic interactions: the modulation of enzyme function by neighboring colloidal ɣ-Fe_2O_3

Graziano Rilievo[1], Massimiliano Magro[1], Federica Tonolo[1], Alessandro Cecconello[1], Lavinia Rutigliano[2], Aura Cencini[1], Simone Molinari[3], Maria L. Di Paolo[4], Cristian Fiorucci[5], Marianna Nicoletta Rossi[5], Manuela Cervelli[5,*], Fabio Vianello[1,6]

1 Department of Comparative Biomedicine and Food Science, University of Padua, Viale dell'Università 16, 35020 Legnaro, Italy
2 Department of Molecular medicine, Sapienza University of Rome, Laboratory Affiliated to Istituto Pasteur Italia, Fondazione Cenci Bolognetti, Sapienza University of Rome, Viale Regina Elena 291, 00161 Rome, Italy
3 Department of Geosciences, University of Padua, Via Gradenigo 6, 35131 Padova, Italy
4 Department of Molecular Medicine, University of Padua, University of Padua, Via G. Colombo 3, 35131 Padova, Italy
5 Department of Science, University of Roma 3, Viale Guglielmo Marconi 446, 00146, Rome, Italy
6 International Polyamines Foundation 'ETS-ONLUS', Via del Forte Tiburtino 98, 00159 Rome, Italy
* Correspondence: manuela.cervelli@uniroma3.it; Tel.: +39 06 5733 82448

Supplementary Information

Supplementary figures

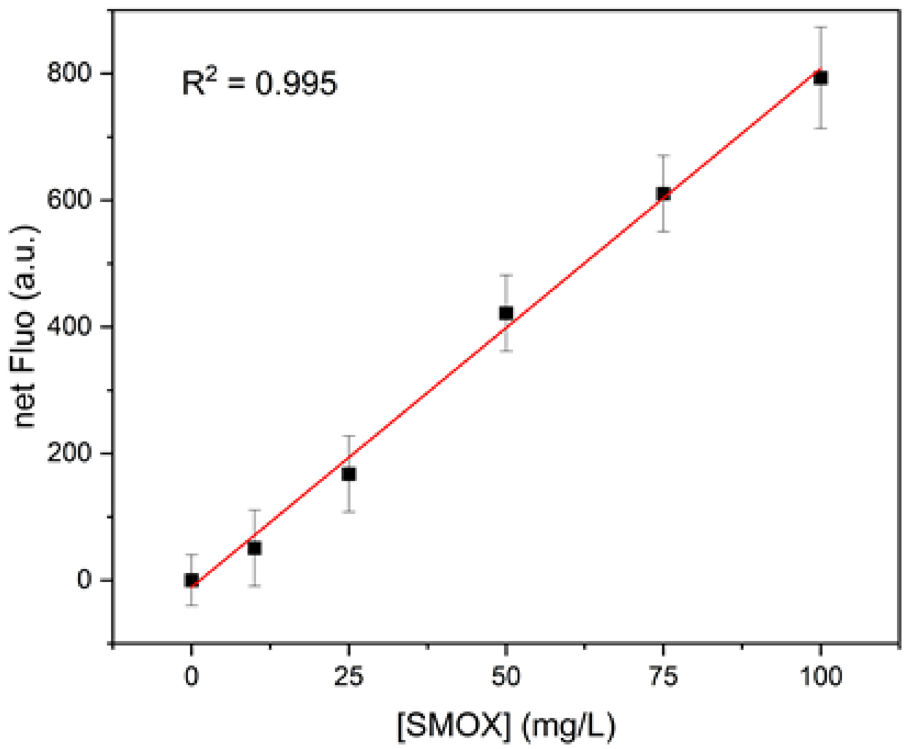

Figure S1. Fluorescence calibration curve of the enzyme spermine oxidase in 10 mM HEPPS pH 8.

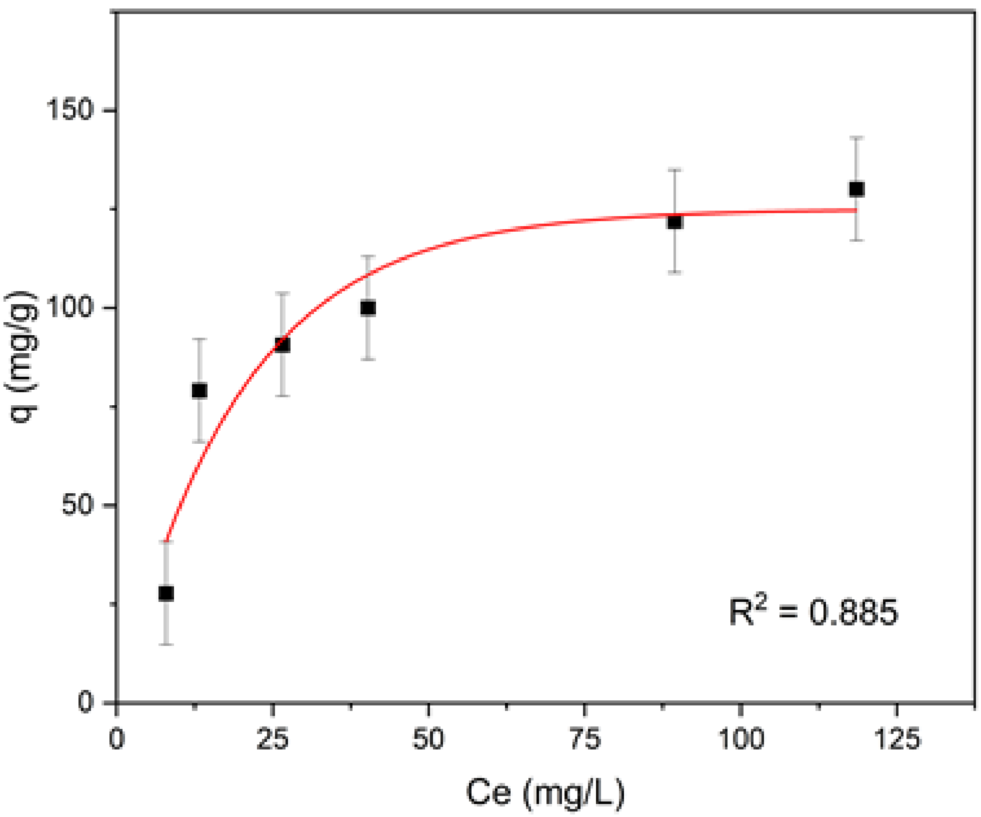

Figure S2. Giles isotherm of the SMOX binding onto SAMNs.

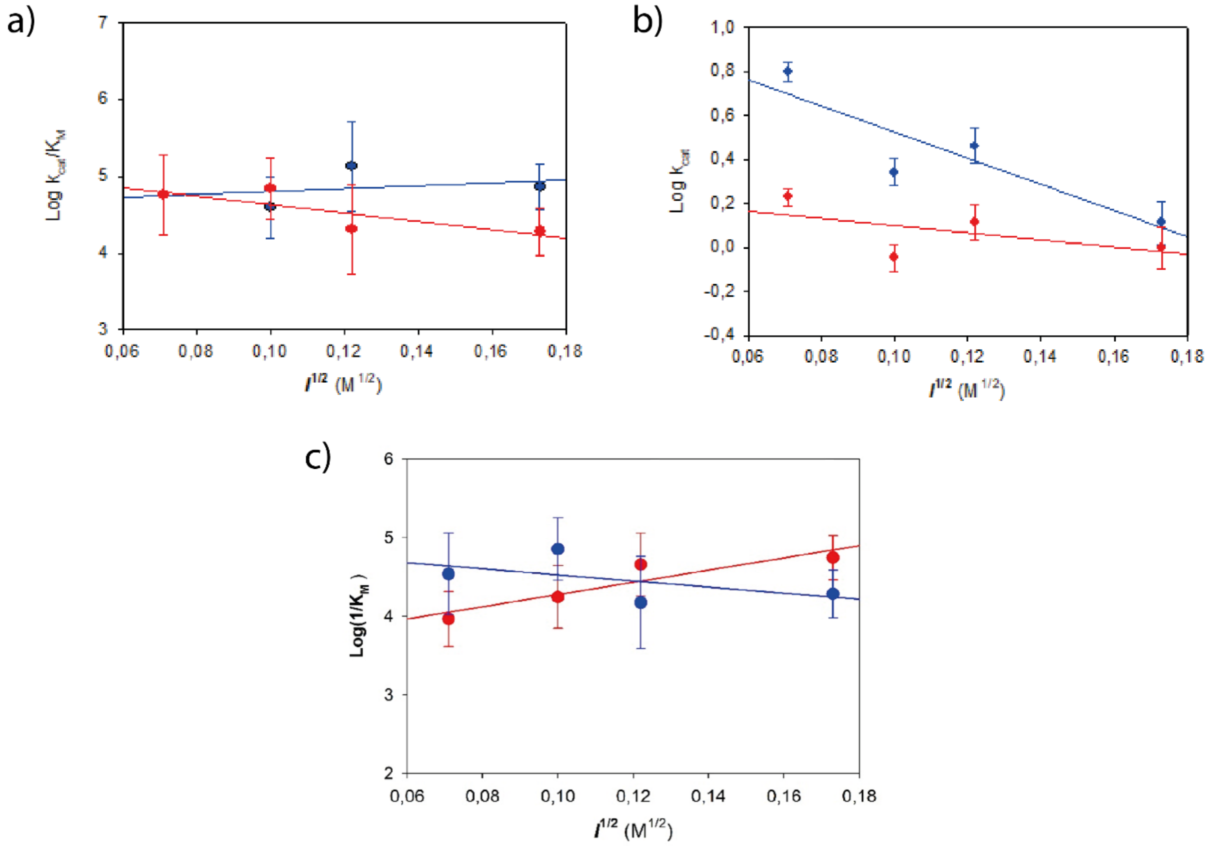

Figure S3. Dependence of kinetic parameters of soluble SMOX and SAMN@SMOX on ionic strength. A) Dependence of Log(k_{cat}/K_M) of SMOX and SAMN@SMOX on ionic strength; B) Dependence of Log(k_{cat}) of SMOX and SAMN@SMOX on ionic strength; C) Dependence of Log(1/K_M) of SMOX and SAMN@SMOX on ionic strength; (●), SMOX; (●), SAMN@SMOX
